# Immunological parameters in girls with Turner syndrome

**DOI:** 10.1186/1477-5751-3-6

**Published:** 2004-11-25

**Authors:** Annika E Stenberg, Lisskulla Sylvén, Carl GM Magnusson, Malou Hultcrantz

**Affiliations:** 1 Dept. of Otorhinolaryngology, Karolinska University Hospital, Stockholm, Sweden; 2 Dept. of Woman and Child Health, Karolinska University Hospital, Stockholm, Sweden; 3 Dept. Clin. Chemistry, Engelholm hospital, Engelholm, Sweden

**Keywords:** Antibodies, lymphocytes, immunoglobulins, hearing, otitis media

## Abstract

Disturbances in the immune system has been described in Turner syndrome, with an association to low levels of IgG and IgM and decreased levels of T- and B-lymphocytes. Also different autoimmune diseases have been connected to Turner syndrome (45, X), thyroiditis being the most common.

Besides the typical features of Turner syndrome (short stature, failure to enter puberty spontaneously and infertility due to ovarian insufficiency) ear problems are common (recurrent otitis media and progressive sensorineural hearing disorder).

Levels of IgG, IgA, IgM, IgD and the four IgG subclasses as well as T- and B-lymphocyte subpopulations were investigated in 15 girls with Turners syndrome to examine whether an immunodeficiency may be the cause of their high incidence of otitis media. No major immunological deficiency was found that could explain the increased incidence of otitis media in the young Turner girls.

## Introduction

Recurrent otitis media is often a problem in children with Turner syndrome (TS) [1,2]. More than 60% of the Turner girls (60–80%) aged 4–15 years suffer from repeated attacks of acute otitis media, as compared to 5% of children (aged 0–6 years) in the normal population [3,4]. These problems among the Turner girls are more extensive and last longer (up in their teens) than in an non Turner population. Frequent insertions of myringeal tubes are often necessary and in order to try to prevent chronic ear problems regular and frequent controls are necessary. However, sequelae like chronic otitis media are frequently seen, even if controls have been meticulous. A sensorineural hearing loss is also common among these patients, with a typical dip in the mid frequencies, declining over time. This sensorineural dip has been identified already in 6-year-old Turner girls [3]. Later in life (~35 years) a progressive high frequency hearing loss is added to the dip, leading to more prominent hearing problems and hearing aids often become necessary [2,5,6]. The cause of the associated ear and hearing problems is not known but the ear problems later in life could be influenced by the loss of estrogen.

TS is caused by the presence of only one normally functioning X-chromosome. The other sex chromosome can be missing (45, X) or abnormal and mosaicism is often present. Occurring in one of every 2000 female births, TS is one of our most common sex chromosome abnormalities [7]. TS is characterized by short stature, no spontaneous puberty and infertility due to ovarian dysgenesis with no estrogen production [8]. Mental retardation is not connected to the syndrome. Since the early 80's, treatment is given with growth hormone from birth and estrogen therapy to induce puberty.

Immunological disturbances have previously been described in TS, with an association to reduced levels of serum IgG and IgM, increased IgA and decreased levels of circulating T- and B-lymphocytes. However, the results have not been conclusive [9-12].

In the normal population children with IgG_2 _deficiency commonly develop recurrent acute otitis media. It is believed that these infections are secondary to impaired antibody response, rather than Eustachian tube dysfunction [13]. As immunological derangements seem to be common in TS, an immunological deficiency could be a potential cause to parts of the ear problems.

The aim of this study was to investigate immunoglobulin and lymphocyte subpopulations in girls with Turners syndrome to examine whether an immunodeficiency may be the cause of their high incidence of otitis media. Immunotherapy would then be a possible treatment.

## Materials and methods

### Subjects

Blood samples from patients with the diagnosis TS, genetically confirmed, were investigated according to the Swedish ethical record no 88–265.

Analyses regarding immunoglobulin- and lymhpocyte subpopulations were performed in 15 girls, aged 5–17 years (median age 11 years), randomly selected from all girls in this age group with TS attending the Karolinska Hospital, Stockholm (total 29 patients). Of these 53% (n = 8) had suffered from repeated attacks of otitis media. All TS girls had been treated with growth hormones and their karyotypes were: 45, X (n = 8); 45, X/46, XX (n = 4); 45, X/46, X, i(Xq) (n = 2); and 45, X/46, X, r(X) (n = 1) (r = ring chromosome).

A medical history was attained, focusing on autoimmune diseases, previous and current ear diseases and other infectious diseases, ear operations, and hearing problems.

### Lymphocyte subpopulations

Leukocyte counts (10^9^/L) were analysed in a Coulter MicroDiff II (Beckman-Coulter). The differential leukocyte (lymphocytes, monocytes and granulocytes) counts and percentages were obtained by 2-color FACS-analysis with CD14/CD45 markers. The number and percentage of lymphocyte subpopulations were obtained by standardized 2- or 3-color FACS-analysis on Epics XL or Elite flowcytometer (Beckman-Coulter) using commercial reagents. CD19^+ ^was marker for B-cells and CD3^+ ^for T-cells, CD3^+^CD4^+ ^for helper T-cells, CD3^+^CD8^+ ^for cytotoxic T-cells, CD56^+^CD3^- ^for NK-cells and HLA-DR^+ ^for activated T-cell subsets. The ratio of CD4^+^/CD8^+ ^was also calculated. The monoclonal antibody clones used were: UCHT1 (CD3^+^), SFCI12T4D11/T4 (CD4^+^), SFCI21Thy2D3/T8 (CD8^+^), 116/Mo2 (CD14^+^), 89B/B4 (CD19^+^), KC56 (CD45^+^), NKH1 (CD56^+^) and 9-49/I3 (HLA-DR^+^), all from Cytostat, Beckman-Coulter. All FACS-analyses were performed at the routine laboratory, Department of Clinical Immunology, Karolinska Hospital and the results were compared to age-related in-house and published reference ranges (5 to 95 percentiles) [14] except for CD56^+^CD3^- ^for which an adult reference was used (10–90 percentile).

### Complement and antibodies

Hemolytic complement (classical and alternative pathways), IgA antibodies to gliadin and endomysium, IgG antibodies to pneumococcal polysaccharide and tetanus toxoid antigen, the serum concentrations (g/L) of circulating IgA, IgG, IgM, IgD, IgG_1_, IgG_2_, IgG_3 _and IgG_4 _as well as the Gm(23)-allotyping of IgG_2 _were analysed by standard methods and compared to age related reference ranges used at the routine laboratory, Department of Clinical Immunology, Karolinska Hospital, Stockholm.

### Statistical analysis

Medians of continuous parameters were compared between groups by Mann-Whitney U-test and correlations were performed by Spearman rank analysis. A two-tailed p < 0.05 was considered significant.

## Results

### Lymphocyte subpopulations

**Figure 1:**
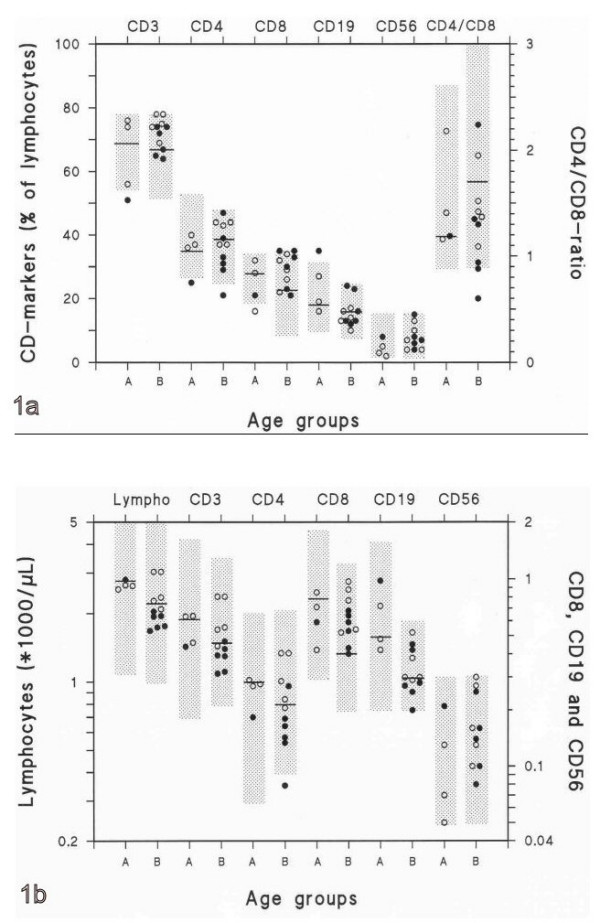
**1a and b **Percentages (Fig 1a) and absolute counts (Fig 1b) of lymphocyte subpopulations in 15 girls with Turner's syndrome divided into two age groups. Group A aged <10 years (n = 4) and group B aged ≥10 years (n = 11). Girls with recurrent otitis media are illustrated with open symbols (n = 8) and those who are otitis free with filled symbols (n = 7). The horizontal lines indicate medians and the shaded boxes the 5 to 95 percentiles of age-related reference ranges except for CD56^+^CD3^- ^cells for which the 10 to 90 percentiles reference range of adults was used.

The leukocyte counts as well as the absolute counts and percentages of lymphocytes, monocytes, and granulocytes were within normal limits for all 15 Turner girls. Likewise most girls had normal counts and percentages of lymphocyte subpopulations as compared to the 5 to 95% percentiles age-related reference ranges (Fig. 1a and 1b) including activated CD4^+ ^and CD8^+ ^T-cells (HLA-DR^+^). However, the CD4^+^/CD8^+ ^ratio was in the lower range (girls aged ≥10), with one girl having a very low ratio (0.6).

### Complement and Immunoglobulin levels

Hemolytic complement (classical and alternative pathway) was within normal limits for all 15 Turner girls.

**Figure 2:**
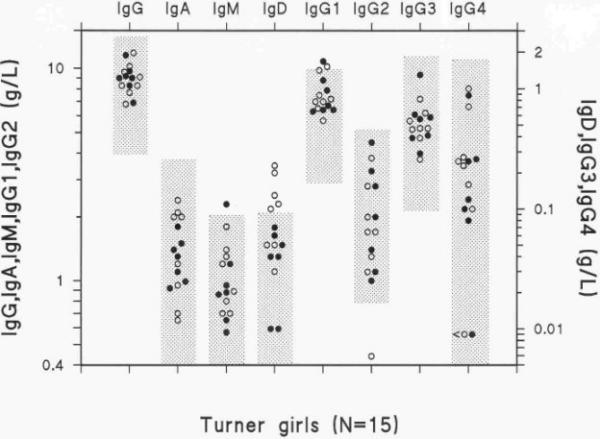
Immunoglobulin levels in 15 Turner girls. The shaded boxes indicate the 95% confidence interval for the 5–20 years age group. Girls with recurrent otitis media are illustrated with open symbols (n = 8) and those who are otitis free with filled symbols (n = 7).

The serum concentrations of IgG, IgA, IgM, IgD and the four IgG subclasses were for most Turner girls within the age-related 95% confidence intervals (Fig. 2). The exceptions were one girl with elevated IgM (2.3 g/L), five with elevated IgD (0.1–0.23 g/L), two with elevated IgG_1 _(10.2 and 10.8 g/L), one with low IgG_2 _(0.4 g/L) and two girls with low IgG_4 _(<0.01 g/L).

The frequency of homozygous G2m(23)-negative Turner girls was 33% (5/15).

### Antibodies

Normal levels of IgG antibodies to tetanus toxoid and polysaccharide antigen were detected among most Turner girls, except for two respectively one, having too low levels. Slightly elevated IgA antibodies to gliadin were observed in 3 (20%) girls, whereas no IgA antibodies to endomysium could be detected in any of the 15 girls.

### Age

When comparing girls aged <10 years (n = 4) and ≥10 years (n = 11) the following parameters were found to be influenced by age with decreased values among the older girls: total counts of leukocytes (p = 0.0093), lymphocytes (p < 0.05), monocytes (p = 0.0093), granulocytes (p = 0.015), CD19^+ ^(p = 0.0053) and CD4^+^HLA-DR^+ ^(p = 0.035), as well as the percentage of CD19^+ ^(p = 0.023). Also IgG_2 _increased with age (p = 0.05). These findings are in line with the reference literature for the normal population [14].

### Recurrent Otitis Media

The girls with TS were divided into two groups according to their history of recurrent otitis media. As age influenced some of the parameters we only considered girls ≥10 years old (n = 11). Significant increases in absolute counts of lymphocytes (p = 0.004), CD3^+ ^T-cells (p = 0.0087), CD4^+ ^T-cells (p = 0.012) and CD4^+^HLA-DR^+ ^(p = 0.05) as well as in the percentage of CD3^+ ^T-cells (p = 0.05) in otitis prone (n = 5) compared to otitis free (n = 6) Turner girls was shown. No such differences were noticed for any immunoglobulin levels, antibody titers, CD4^+^/CD8^+^-ratio or CD8^+^, CD19^+^, CD56^+^CD3^- ^lymphocyte subpopulations.

### Karyotype

Any apparent influence, of the different karyotypes, on any of the parameters studied was not observed within the group.

## Discussion

In this study no major derangement in the immune status was found among the girls with TS. Normal levels of most lymphocyte- and immunoglobulin subpopulations were registered. The few outliers noted must be considered as a normal individual variation.

However, as described in an earlier study of Turner girls, the present study confirmed a CD4^+^/CD8^+ ^ratio in the lower range [12], supposedly as a consequence of a slightly increased CD8^+ ^population. Although, the patients were few, we noticed some differences between the otitis prone and otitis free Turner girls. The elevated counts of lymphocytes, CD3^+^, CD4^+ ^cells and CD4^+^HLA-DR^+ ^cells seen among the otitis prone girls, probably reflects a secondary effect of an activated immune system involving T-helper cells, rather than any immune deficient state. Moreover, the levels of IgG antibodies to pneumococcal polysaccharide antigen, which are important in the defense of bacteria, were normal. A homozygous lack of the IgG2m(23) allotype was seen in 33% of the girls, which is the same frequency as in the normal population [15]. A negative IgG2m(23) allotype have been correlated to an impaired immune response to haemophilus influenzae vaccination with subnormal levels of IgG_2_. In the study group a negative IgG2m(23) allotype was not correlated to a positive history of recurrent otitis media, neither could the different karyotypes be associated to the levels of immunoglobulin- or lymphocyte subpopulations. Perhaps the cause of the repeated attacks of otitis media in Turners syndrome is not to be found in the periphery, but rather more locally. Even if earlier computed tomography scans of the temporal bone have not shown any abnormalities [2], the Eustachian tube may be dysfunctional and/or the cell system might be underdeveloped. Recently new aspects on the growth of the temporal bone have been proposed, with a hypothesis that the loss of X-chromosome material leads to a prolonged cell cycle and otic growth disturbances during fetal life [16]. The SHOX-gene located on the p-arm of the X-chromosome has been found to code for growth and could potentially also code for growth of the skull base and temporal bone where the middle ear is located. [17]. As the girls investigated were 5–17 years old, transient hypogammaglobulinemia in the first years is still possible. However, the girls suffered otitis media up in their teens.

Our findings of normal immunoglobulin- and lymphocyte subpopulations are not entirely in concordance with some earlier studies, where a reduction of circulating IgM and IgG as well as T- and B-lymphocytes has been observed [9,10]. However, in these studies the values were not dramatically decreased, but rather within the lower range of the normal reference values. On the other hand, some other studies have not shown low T- and B-lymphocyte counts [11] or low concentrations of immunoglobulins [12], agreeing with the present study. In the normal population there is a difference between IgG and IgM levels in women and men with decreased values in men [12], but this difference cannot be found in newborns or children. Earlier there have been suggestions that the difference is caused by the amount of X chromosome material, as men with 47, XXY have higher values than men with normal karyotype (46, XY) and women with 47, XXX have even higher values than normal women (46, XX) [18]. There have also been suggestions that the sex hormones influence the immune system and that the lack of estrogens might influence the immune response negatively [11]. As most of the girls studied were prepubertal, the influence from sex hormones should not be as important. In some earlier studies the age span has been wider and the size of the study groups relatively small. There have also been discussions that the regular treatment with growth hormones may influence the immune system. However, in a previous study no major effects on the immunoglobulin levels or lymphocyte subpopulations could be demonstrated in Turner girls treated with growth hormones [12].

In conclusion, we did not find any major immunological deficiency in immunoglobulins or lymphocyte subpopulations that could explain the increased incidence of otitis media observed in girls with TS. Therefore, treatment with immunotherapy is not an option in this patient group. Further studies are warranted to elucidate local pathology, both from an immunological and anatomical point of view.

## Authors' contributions

AES participated in the design of the study, performed the statistical analysis and drafted the manuscript. LS participated in the design of the study and collected the blood samples. CGMM performed the statistical analysis. MH participated in the design and coordination of the study and collected the blood samples.

All authors read and approved the final manuscript.
